# Electron cooling in graphene enhanced by plasmon–hydron resonance

**DOI:** 10.1038/s41565-023-01421-3

**Published:** 2023-06-22

**Authors:** Xiaoqing Yu, Alessandro Principi, Klaas-Jan Tielrooij, Mischa Bonn, Nikita Kavokine

**Affiliations:** 1 Max Planck Institute for Polymer Research, Mainz, Germany; 2 School of Physics and Astronomy, University of Manchester, Manchester, UK; 3 Catalan Institute of Nanoscience and Nanotechnology (ICN2), BIST and CSIC, Campus UAB, Bellaterra, Barcelona Spain; 4 Department of Applied Physics, TU Eindhoven, Eindhoven, Netherlands; 5 Center for Computational Quantum Physics, Flatiron Institute, New York, NY USA

**Keywords:** Electronic properties and devices, Surfaces, interfaces and thin films

## Abstract

Evidence is accumulating for the crucial role of a solid’s free electrons in the dynamics of solid–liquid interfaces. Liquids induce electronic polarization and drive electric currents as they flow; electronic excitations, in turn, participate in hydrodynamic friction. Yet, the underlying solid–liquid interactions have been lacking a direct experimental probe. Here we study the energy transfer across liquid–graphene interfaces using ultrafast spectroscopy. The graphene electrons are heated up quasi-instantaneously by a visible excitation pulse, and the time evolution of the electronic temperature is then monitored with a terahertz pulse. We observe that water accelerates the cooling of the graphene electrons, whereas other polar liquids leave the cooling dynamics largely unaffected. A quantum theory of solid–liquid heat transfer accounts for the water-specific cooling enhancement through a resonance between the graphene surface plasmon mode and the so-called hydrons—water charge fluctuations—particularly the water libration modes, which allows for efficient energy transfer. Our results provide direct experimental evidence of a solid–liquid interaction mediated by collective modes and support the theoretically proposed mechanism for quantum friction. They further reveal a particularly large thermal boundary conductance for the water–graphene interface and suggest strategies for enhancing the thermal conductivity in graphene-based nanostructures.

## Abstract (web summary)

Ultrafast spectroscopy experiments demonstrate that graphene electrons can transfer energy directly to liquid water with no mediation from the crystal lattice as their collective plasmon oscillation excites water’s molecular charge fluctuations.

## Main

Free electrons in graphene exhibit rather unique dynamics in the terahertz frequency range, including a highly nonlinear response to photoexcitation by terahertz pulses^1,2^. Graphene’s distinctive dynamic properties on picosecond time scales have found several applications in, for example, ultrafast photodetectors, modulators and receivers^3–5^. The terahertz frequency range acquires particular importance at room temperature *T*, where it corresponds to the typical frequency of thermal fluctuations: *k*_B_*T*/*ℏ* ≈ 6 THz, where *k*_B_ is Boltzmann’s constant and *ℏ* is Planck’s constant. One may therefore expect non-trivial couplings between the graphene electrons and the thermal fluctuations of their environment. These couplings have been intensively studied in the case of a solid environment: for instance, non-adiabatic effects have been shown to arise in the graphene electron–phonon interaction^6^, and plasmon–phonon coupling between graphene and a polar substrate has been demonstrated^7–9^. More recently, it has been theoretically proposed that similar effects are at play when graphene has a liquid environment: then, the interaction between the liquid’s charge fluctuations—dubbed hydrons—and graphene’s electronic excitations tunes the hydrodynamic friction at the carbon surface^10,11^. This ‘quantum friction’ mechanism holds the potential for entirely new strategies for controlling liquid flows on the nanometre scale^12,13^; it is therefore of interest to experimentally probe the underlying electron–hydron interaction.

**Fig. 1:**
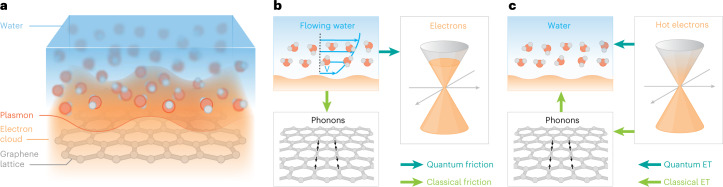
Heat transfer and friction at the solid–liquid interface. **a**, Schematics of the system under study: the interface between water and a graphene sheet. The picture emphasizes the electron cloud and its wave-like plasmon excitation. **b**, Momentum transfer processes at the solid–liquid interface. A flowing liquid (the flow profile is shown by the thin blue arrows) may not only transfer momentum to the crystal lattice (exciting phonon vibrations) through classical hydrodynamic friction, but also directly to the electrons through quantum friction. **c**, Energy transfer (ET) processes at the solid–liquid interface. In the typically assumed ‘classical’ pathway, hot electrons first transfer energy to the phonons, which transfer energy to the liquid. An alternative ‘quantum’ pathway consists in the electrons transferring energy directly to the liquid through Coulomb coupling.

In this article, we probe solid–liquid interactions by measuring energy transfer at the solid–liquid interface (Fig. 1a). Specifically, we use a femtosecond visible pulse to introduce a quasi-instantaneous temperature difference between the electrons of a graphene sample and their environment. The cooling rate of the electronic system is followed in real-time using terahertz pulses. Such optical pump–terahertz probe spectroscopy is a well-established tool for probing electron relaxation in two-dimensional materials^14–19^. In high-quality graphene, it has been used to identify the interaction of hot carriers with optical phonons^17,18^ and with substrate phonons as the main electron cooling mechanisms^20^; it has also identified the role of Coulomb interactions in the interlayer thermal conductivity of graphene stacks^16^. Here, we measure the electron relaxation time in the presence of different polar liquids to probe the electron–hydron interaction, which we find to be comparable to the electron–optical phonon interaction only when the liquid is water. A complete theoretical analysis shows that this specificity of water is explained by the strong coupling of its terahertz (libration) modes to the graphene surface plasmon, with the electron–electron interactions in graphene playing a crucial role.

## Solid–liquid heat transfer

The energy transfer between a solid and a liquid is usually considered to be mediated by molecular vibrations at the interface because most of a solid’s heat capacity is contained in its phonon modes^21^. Even if an optical excitation of the solid’s electrons is used to create the temperature difference, the electrons are typically assumed to thermalize with phonons on a very short time scale, so that the solid’s phonons ultimately mediate the energy transfer to the liquid’s vibrational modes^22,23^. However, if the electrons were to transfer energy to the liquid faster than to the phonons, the interfacial thermal conductivity would contain a non-negligible contribution from near-field radiative heat transfer^24,25^ (Fig. 1c). Such an electronic or ‘quantum’ contribution to heat transfer is in close analogy with the quantum contribution to hydrodynamic friction. Quantum hydrodynamic friction relies on momentum being transferred directly between the solid’s and the liquid’s charge fluctuation modes, coupled by Coulomb forces (Fig. 1b): the two processes are mediated by the same solid–liquid interaction.

To probe this interaction through a hydrodynamic friction measurement, one needs to ensure that quantum friction dominates over the classical surface roughness contribution: this imposes stringent constraints on the sample’s surface state, in already technically difficult experiments^26–28^. Similarly, in the case of energy transfer, the quantum contribution needs to be comparable to the classical phonon-based contribution to become measurable; however, this condition is easier to satisfy since it is insensitive to the sample’s surface roughness. We show that this condition is met upon optically exciting a graphene–water interface, due, in particular, to graphene’s weak electron–phonon coupling^29,30^.

## Time-resolved electron cooling

**Fig. 2:**
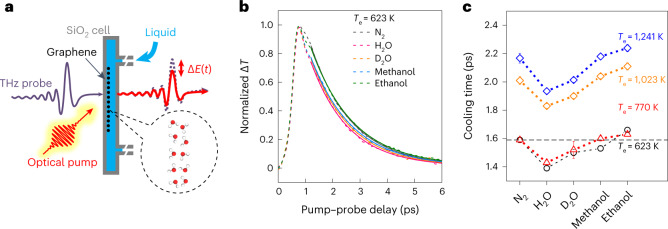
Measurement of picosecond hot electron relaxation in graphene. **a**, Schematic of the experimental set-up. A graphene sample (Fermi level in the range 100−180 meV; Supplementary Information, section 1.4) is placed in contact with a liquid inside a fused silica flow cell. An optical excitation pulse quasi-instantaneously heats up the graphene electrons, and the electron temperature dynamics are then monitored with a THz probe. **b**, Normalized electron temperature as a function of time after photoexcitation. The dotted lines represent raw data and the full lines are exponential fits. **c**, Electron cooling time obtained through exponential fitting (see **b**) for the different liquids that have been placed in the flow cell and different initial electron temperatures, set by the excitation laser fluence. Faster cooling is observed in the presence of water and heavy water. Error bars represent 95% confidence intervals of the exponential fits, and the centre point corresponds to the result of the least-squares fitting procedure.

Our experimental set-up is schematically represented in Fig. 2a. A monolayer graphene sample grown by chemical vapour deposition (CVD) was transferred onto a fused silica flow cell, filled with either nitrogen gas or a liquid of our choice (Supplementary Information, section 1.1). The graphene chemical potential was in the range 100–180 meV as determined from Raman measurements (Supplementary Information, section 1.4). In a typical experiment, the graphene electrons were excited using an ~50 fs laser pulse with 800 nm central wavelength. Then, the attenuation of an ~1 ps THz probe pulse (precisely, the modulation of the peak electric field) was monitored as a function of the pump–probe delay (Supplementary Information, section 1.2). After absorption of the exciting pump pulse, the non-equilibrium electron distribution typically thermalizes over a sub-100 fs time scale through electron–electron scattering^31^: it can then be described as a Fermi–Dirac distribution at a given temperature. A hotter electron distribution results in a lower terahertz photoconductivity because hotter electrons are less efficient at screening charged impurities^32,33^. The pump–probe measurement thus gives access to the electron temperature dynamics after photoexcitation (Fig. 2b).

Regardless of the medium that the graphene is in contact with, the electronic temperature *T*(*t*) exhibits a relaxation that can be approximated by an exponential function: Δ*T*(*t*) = *T*(*t*) − *T*_0_ = Δ*T*_0_*e*^−*t*/*τ*^. This allows us to extract the cooling times *τ* for the different liquids and different initial electronic temperatures (determined by the excitation laser fluence), displayed in Fig. 2c. We observe that the cooling time is longer for an initially hotter electron distribution, in agreement with previous reports^18^. Now, for all initial temperatures, we consistently observe the same dependence of the cooling time on the sample’s liquid environment. In the presence of water (H_2_O) and heavy water (D_2_O), the graphene electrons cool faster than they do intrinsically, in an inert nitrogen atmosphere. Conversely, methanol and ethanol have almost no effect on the electron cooling time. Interestingly, we observe an isotope effect in the electron cooling process: there is a difference in the cooling times in the presence of H_2_O and D_2_O that greatly exceeds experimental uncertainties.

We are thus led to hypothesize, as anticipated above, that the liquid provides the electrons with a supplementary cooling pathway, which, in the case of water, has an efficiency comparable to the intrinsic cooling pathway via phonons. We then interpret the faster cooling as a signature of ‘quantum’ electron–liquid energy transfer. We assess the pertinence of this hypothesis by developing a complete theory of quantum energy transfer at the solid–liquid interface.

## Theoretical framework

To tackle the interaction between a classical liquid and an electronic system whose behaviour is intrinsically quantum, we describe the liquid in a formally quantum way. Following ref. ^10^, we represent the liquid’s charge density as a free fluctuating field with prescribed correlation functions. This naturally leads to a Fourier-space description of the solid–liquid interface in terms of its collective modes, rather than the usual molecular-scale interactions. Within this description, the quantum solid–liquid energy transfer amounts to electron relaxation upon coupling to a bosonic bath, a problem that has been extensively studied in condensed matter systems^34^. Interestingly, in the case of graphene, many of these studies are carried out within a single-particle Boltzmann formalism, which may incorporate multiple screening effects only in an ad hoc fashion^18,29,35^. These effects turn out to be crucial for the solid–liquid system under consideration: we have therefore developed an ab initio theory of solid–liquid heat transfer based on the non-equilibrium Keldysh formalism^36^, which has only very recently been considered for problems of interfacial heat transfer^37^. Our computation, detailed in Supplementary Information, section 2.2, is closely analogous to the one carried out for quantum friction in ref. ^10^. The theoretical framework can formally apply to fully non-equilibrium situations and take interactions into account to arbitrary order. However, to obtain a closed-form result, assume that the liquid and the solid internally equilibrated at temperatures *T*_l_ and *T*_e_, respectively. Furthermore, we take electron–electron and electron–liquid Coulomb interactions into account at the random phase approximation (RPA) level. With these assumptions, we obtain the electron–liquid energy transfer rate as

1 $$\begin{array}{l}{{{{\mathcal{Q}}}}}_{{{{\rm{Q}}}}}=\dfrac{1}{2{\uppi }^{3}}{\displaystyle{\int}}{{{\rm{d}}}}{{{\bf{q}}}}\mathop{\displaystyle{\int}}\nolimits_{0}^{+\infty }{{{\rm{d}}}}\omega \,\hslash \omega \left[\right.{n}_{{{{\rm{B}}}}}(\omega ,{T}_{{{{\rm{e}}}}})\\\qquad\;-{n}_{{{{\rm{B}}}}}(\omega ,{T}_{\rm{l}})\left.\right]\dfrac{{{{\rm{Im}}}}\,[{g}_{{{{\rm{e}}}}}({{{\bf{q}}}},\omega )]{{{\rm{Im}}}}\,[{g}_{{{{\rm{l}}}}}({{{\bf{q}}}},\omega )]}{| 1-{g}_{{{{\rm{e}}}}}({{{\bf{q}}}},\omega ){g}_{{{{\rm{l}}}}}({{{\bf{q}}}},\omega ){| }^{2}},\end{array}$$

Here, *n*_B_(*ω*,*T*) = 1/(*e*^*ℏω*/*T*^ − 1) is the Bose distribution and the *g*_e,l_ are surface response functions of the solid and the liquid, respectively. These are analogues of the dielectric function for semi-infinite media, the precise definition of which is given in Supplementary Information, section 2.3. For the liquids under consideration, it will be sufficient to use the long-wavelength-limit expression of the surface response function:

2 $${g}_{{{{\rm{l}}}}}(q\to 0,\omega )=\frac{{\epsilon }_{\rm{l}}(\omega )-1}{{\epsilon }_{\rm{l}}(\omega )+1},$$

where *ε*_l_(*ω*) is the liquid’s bulk dielectric function. For two-dimensional graphene, we show in Supplementary Information, section 2.3 that the surface response function can be expressed as

3 $${g}_{{{{\rm{e}}}}}(q,\omega )=-\frac{{e}^{2}}{2{\epsilon }_{0}q}\chi (q,\omega ),$$

where *χ*(*q*,*ω*) is graphene’s charge susceptibility.

The result in equation (1) has been derived for two solids separated by a vacuum gap in the framework of fluctuation-induced electromagnetic phenomena^24,38,39^; our non-equilibrium framework, however, is better suited to the solid–liquid system under consideration. We note that equation (1) takes the form of a Landauer formula for the transport of bosonic quasiparticles—elementary excitations of the solid’s and the liquid’s charge fluctuations modes^25^. It involves the difference in the Bose distribution functions between the solid and the liquid, and the product of surface response functions plays the role of a transmission coefficient for the quasiparticles. One may count either the energy or the momentum transported by the quasiparticles: the former corresponds to near-field heat transfer, the latter to quantum friction. This quasiparticle picture thus makes explicit the fundamental connection between the two processes.

## Plasmon–hydron resonance

**Fig. 3:**
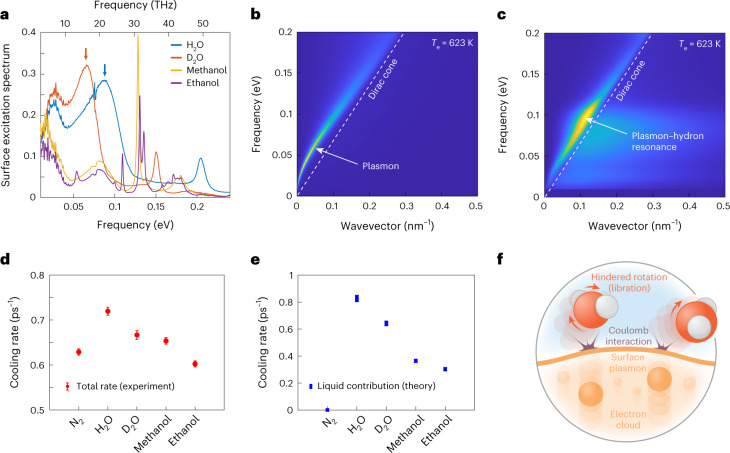
Mechanism of electron–liquid heat transfer. **a**, Surface excitation spectra ${{{\rm{Im}}}}\,[{g}_{\rm{l}}(\omega )]$ of the different liquids studied here obtained according to equation (2) from the experimentally measured bulk dielectric permittivities. The arrows indicate the libration modes of H_2_O and D_2_O. **b**, Graphene surface excitation spectrum ${{{\rm{Im}}}}\,[{g}_{{{{\rm{e}}}}}(q,\omega )]$, calculated at a chemical potential *μ* = 100 meV and temperature *T*_e_ = 623 K. The main feature is the collective plasmon mode. **c**, Theoretical prediction for the graphene–water energy transfer rate resolved in frequency–wavevector space. The main contribution originates from a resonance between the graphene plasmon mode and the water libration mode. **d**, Experimentally measured electron cooling rate in the presence of the various liquids, for an initial electron temperature *T*_e_ = 623 K. Error bars represent 95% confidence intervals of the exponential fits to the temperature decay curves. **e**, Theoretical prediction for the liquid contribution to the electron cooling rate, reproducing the experimentally observed trend in terms of the nature of the liquid. The symbol size in the vertical direction represents the variation in the theoretical prediction when the graphene chemical potential spans the range 100–180 meV. **f**, Schematic of the water-mediated electron cooling mechanism inferred from the combination of theoretical and experimental results. The cooling proceeds through the Coulomb interaction between the graphene plasmon mode and the hindered molecular rotations (librations) in water.

The graphene electrons may relax either through direct interaction with the liquid, or through emission of optical phonons. The latter process has been well studied, both theoretically and experimentally^18,29^. Our non-equilibrium formalism applies in principle to any electron–boson system: when applied to the electron–phonon system, it recovers the result for the energy transfer rate ${{{{\mathcal{Q}}}}}_{{{{\rm{ph}}}}}$ (from electrons to phonons) obtained in ref. ^18^ (Supplementary Information, section 2.3.2). Then, within a three-temperature model, where the electrons, liquid and phonons are assumed to be internally equilibrated at temperatures *T*_e_, *T*_l_ and *T*_ph_, respectively, we may determine the evolution of the electron temperature according to

4 $$C({T}_{{{{\rm{e}}}}})\frac{{{{\rm{d}}}}{T}_{{{{\rm{e}}}}}(t)}{{{{\rm{d}}}}t}=-{{{{\mathcal{Q}}}}}_{{{{\rm{Q}}}}}({T}_{{{{\rm{e}}}}},{T}_{\rm{l}})-{{{{\mathcal{Q}}}}}_{{{{\rm{ph}}}}}({T}_{{{{\rm{e}}}}},{T}_{{{{\rm{ph}}}}}),$$

where *C*(*T*_e_) is the graphene electronic heat capacity at temperature *T*_e_. We focus in the following on the liquid contribution to the electron cooling rate, defined as $1/\tau ={{{{\mathcal{Q}}}}}_{{{{\rm{Q}}}}}({T}_{{{{\rm{e}}}}},{T}_{\rm{l}})/(C({T}_{{{{\rm{e}}}}})\times ({T}_{{{{\rm{e}}}}}-{T}_{\rm{l}}))$, which may be compared with the experimental results. The quantitative evaluation of *τ* requires the surface response functions of graphene and of the various liquids. We compute the graphene surface response function according to equation (3) by numerical integration^40^ at the chemical potential determined for our samples by Raman spectroscopy (Supplementary Information, section 1.4). For the liquids, we use the expression in equation (2), with the bulk dielectric function determined by infrared absorption spectroscopy (Fig. 3a and Supplementary Information, section 1.3).

Our theoretical prediction for the contribution of the various liquids to the electron cooling rate is shown in Fig. 3e. Quantitatively, we obtain cooling rates of the order of 1 ps^−1^, in excellent agreement with the experimentally observed range (Fig. 3d): our theory indicates that the quantum electron–liquid cooling is a sufficiently efficient process to compete with the intrinsic phonon contribution, estimated at around 0.6 ps^−1^ from the cooling rate in the absence of liquid. Moreover, our theory reproduces the experimentally observed trend in cooling rates, with a significant liquid contribution arising only for water and heavy water; the dependence of the cooling rate on initial electron temperature is also well reproduced (Supplementary Fig. 7). Finally, the theory reproduces the isotope effect, that is, the slightly slower cooling observed with D_2_O as compared with H_2_O.

We may now exploit the theory to gain insight into the microscopic mechanism of the liquid-mediated cooling process. In equation (1), the difference of Bose distributions decreases exponentially at frequencies above *T*_e_/*ℏ* ≈ 100 meV. At frequencies below 100 meV, the graphene spectrum is dominated by a plasmon mode that corresponds to the collective oscillation of electrons in the plane of the graphene layer^40^ (Fig. 3b). In this same frequency range, water and heavy water have a high spectral density due to their libration modes that correspond to hindered molecular rotations^41^ (Fig. 3a). As a result, the energy transfer rate resolved in frequency–momentum space (the integrand in equation (1), plotted in Fig. 3c) has its main contribution from the spectral region where the two modes overlap. We conclude that the particularly efficient electron–water cooling is due to a resonance between the graphene plasmon mode and the water libration modes. This conclusion is further supported by the isotope effect. Indeed, the libration of the heavier D_2_O is at slightly lower frequency than that of the lighter H_2_O, and a higher-frequency mode makes a larger contribution to the cooling rate due to the factor *ℏω* in equation (1). In the Landauer picture, the quasiparticle transport rates are almost the same for the graphene–H_2_O and graphene–D_2_O systems, but in the case of H_2_O each quasiparticle carries more energy. Overall, our experiments evidence a direct interaction between the graphene plasmon and water librations, as shown schematically in Fig. 3f. We note that plasmons have been shown to play a role in the energy transfer between two graphene sheets^42^; however, a plasmon–hydron interaction does not appear to have been suggested as a possible electron relaxation mechanism.

## Interactions and strong coupling

**Fig. 4:**
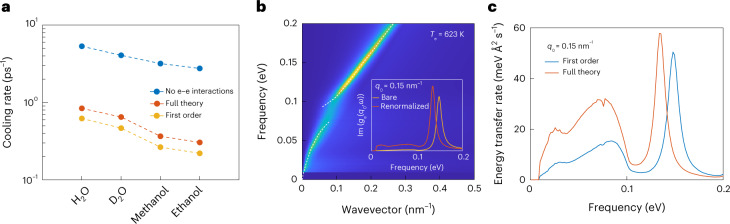
Strong plasmon–hydron coupling. **a**, Theoretical prediction for the graphene electron cooling rate in contact with different liquids, within different treatments of interactions. The cooling rate is strongly overestimated if no electron–electron interactions are taken into account (blue symbols), and underestimated if the electron–liquid interactions are considered only to first order (orange symbols). **b**, Graphene surface excitation spectrum ${{{\rm{Im}}}}\,[{g}_{{{{\rm{e}}}}}(q,\omega )]$, calculated at a chemical potential *μ* = 180 meV and temperature *T*_e_ = 623 K, renormalized by the presence of water according to equation (5). The white dashed lines are guides to the eye showing the strongly coupled plasmon–hydron mode. Inset: bare and renormalized graphene spectra at fixed wavevector *q*_0_ = 0.15 nm^−1^. **c**, Comparison between the spectrally resolved energy transfer rates obtained to first order and to arbitrary order in the solid–liquid interaction. Higher-order effects enhance the energy transfer rate at low frequencies.

The combination of theory and experiment allows us to identify the key physical ingredients that are required to account for energy transfer at the water–graphene interface. First, our results reveal that electron–electron interactions are crucial because they produce the plasmon mode that is instrumental to the energy transfer mechanism. Indeed, applying our theory to non-interacting graphene would result in a strongly overestimated liquid contribution to the cooling rate (Fig. 4a). This precludes single-particle Boltzmann approaches—such as those that have been used for the electron–phonon interaction in graphene^18,29^—from accurately describing the water–graphene interaction.

Furthermore, detailed examination of our theoretical result reveals that the efficiency of the electron–water cooling is enhanced by the formation of a strongly coupled plasmon–hydron mode. Indeed, the result in equation (1) involves bare surface response functions, without any renormalization due to the presence of the other medium. However, the denominator ∣1 − *g*_e_ *g*_l_∣^2^ accounts for solid–liquid interactions to arbitrary order (at the RPA level) and contains the signature of any potential strong coupling effects. We find that these effects are indeed important: removing the denominator in equation (1) (that is, treating the electron–liquid interactions only to first order) results in underestimation of the liquid-mediated cooling rate by about 30% (Fig. 4a). To gain physical insight into the nature of these higher-order effects, we can compute the graphene surface response function renormalized by the presence of water, which is given by (Supplementary Information, section 2.3)

5 $${\tilde{g}}_{{{{\rm{e}}}}}(q,\omega )=\frac{{g}_{{{{\rm{e}}}}}(q,\omega )}{1-{g}_{{{{\rm{e}}}}}(q,\omega ){g}_{\rm{l}}(q,\omega )}.$$

The renormalized surface excitation spectrum ${{{\rm{Im}}}}\,[{\tilde{g}}_{{{{\rm{e}}}}}(q,\omega )]$ is plotted in Fig. 4b for a chemical potential *μ* = 180 meV. We observe that the graphene plasmon now splits into two modes, which are both a mixture of the the bare plasmon and water librations. These are in fact analogous to the coupled plasmon–phonon modes that have been predicted^7^ and measured^8,9^ for graphene on a polar substrate. It can be seen in the inset of Fig. 4b that coupling to the water modes also increases the spectral density at low frequencies (below the plasmon peak) compared with the bare graphene response function. This is in fact the higher-order effect that is mainly responsible for the enhancement of the electron cooling rate. As shown in Fig. 4c, taking into account solid–liquid interactions to arbitrary order mainly enhances the contribution of low frequencies to the energy transfer.

## Conclusions

We have carried out ultrafast measurements of electron relaxation in graphene, revealing signatures of direct energy transfer between the graphene electrons and the surrounding liquid. These results speak to the importance of electronic degrees of freedom in the dynamics of solid–liquid interfaces, particularly interfaces between water and carbon-based materials. Despite conventional theories and simulations that describe the interface in terms of atomic-scale Lennard–Jones potentials^22,23^, or with electronic degrees of freedom in the Born–Oppenheimer approximation^43,44^, here we demonstrate experimentally that the dynamics of the water–graphene interface need to be considered at the level of collective modes in the terahertz frequency range. In particular, our semiquantitative theoretical analysis attributes the observed cooling dynamics to the strong coupling between the graphene plasmon and water libration modes.

The experimental observation of such a collective mode interaction supports the proposed mechanism for quantum friction at the water–carbon interface, which is precisely based on momentum transfer between collective modes^10^. The near-quantitative agreement between the experiment and theory obtained for energy transfer suggests that a similar agreement should be achieved for momentum transfer. The water–graphene quantum friction force is small if the graphene electrons are at rest, but becomes important if they are driven at a high velocity by a phonon wind or an applied voltage^12^. The quantum-friction-based driving of water flows by graphene electronic currents appears as a promising avenue in light of our findings. The electric circuit configuration would furthermore allow for noise thermometry^45,46^ to be used as a supplementary probe of the electron relaxation mechanisms.

Our results provide yet another example of the water–carbon interface outperforming other solid–liquid systems^47^. Indeed, the electronic contribution to the graphene–water thermal boundary conductance is as high as *λ* = 0.25 MW m^−2^ K^−1^, exceeding the value obtained with the other investigated liquids by at least a factor of 2. This even exceeds the thermal boundary conductance obtained for the graphene–hBN interface, at which particularly fast ‘super-Planckian’ energy transfer was observed^20,35^. Our investigation thus suggests that the density of modes in the terahertz frequency range is a key determinant for the thermal conductivity of graphene-containing composite materials.

## Online content

Any methods, additional references, Nature Portfolio reporting summaries, source data, extended data, supplementary information, acknowledgements, peer review information; details of author contributions and competing interests; and statements of data and code availability are available at 10.1038/s41565-023-01421-3.

## Supplementary information

Supplementary Information Supplementary Figs. 1–7, experimental methods and theoretical methods.

## Data Availability

The experimental data supporting the findings are available on Zenodo: 10.5281/zenodo.7738429
