# Supplementary material for: Electron cooling in graphene enhanced by plasmon–hydron resonance
Source: Nat Nanotechnol. 2023 Jun 22;18(8):898–904. doi: 10.1038/s41565-023-01421-3 (PMC10427419; doi:10.1038/s41565-023-01421-3)
Supplement: Supplementary file 1 — Supplementary Figs. 1–7, experimental methods and theoretical methods. [file 41565_2023_1421_MOESM1_ESM.pdf]

nature nanotechnology

Article https://doi.org/10.1038/s41565-023-01421-3

Electron cooling in graphene enhanced by plasmon–hydron resonance

In the format provided by the authors and unedited

Contents

1 Experimental methods **1**
1.1 Sample preparation 1
1.2 OPTP measurements 1
1.3 FTIR measurements 3
1.4 Raman measurements 5

2 Theoretical methods **5**
2.1 Interaction Hamiltonian 6
2.2 General theory of electron-boson heat transfer 7
2.3 Application to the graphene-liquid system 8

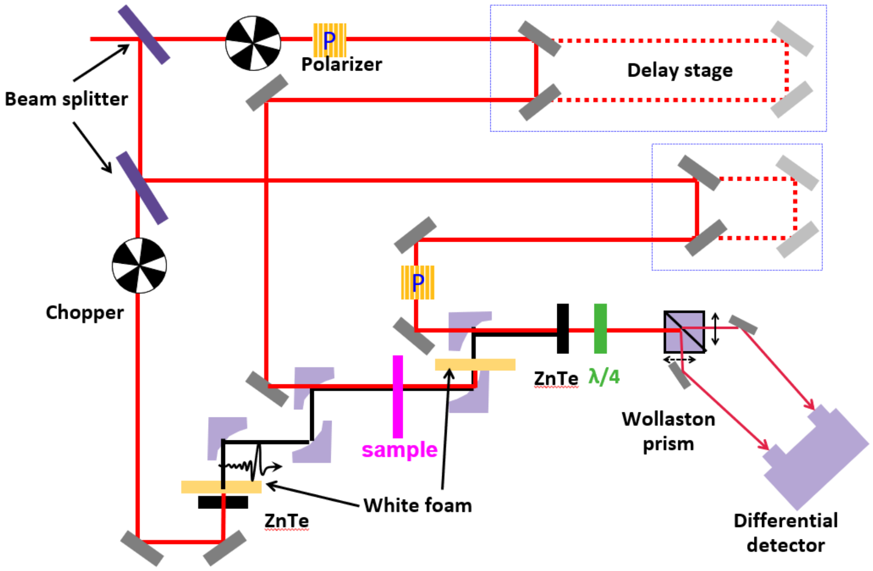

FIGURE S1: Schematic of the OPTP setup.

1 Experimental methods

1.1 Sample preparation

CVD-grown graphene samples supported on 1 mm-thick copper substrates were purchased from Grolltex Inc. The MilliQ water (18.2 MΩ · cm) was used as obtained from the machine. Cellulose acetate butyrate (CAB, average Mn ∼ 12000, Sigma-Aldrich), ammonium persulfate (APS, ACS reagent, ≥ 98%, Honeywell Fluka^{TM}) are used as received. CAB was dissolved in ethyl acetate (Sigma-Aldrich), producing a 30 mg/mL solution. APS was dissolved in MilliQ water to prepare 1 M and 0.1 M solutions. The detachable fused silica flow cell was ordered from FireflySci, Inc. The flow cell was cleaned by sonication in a hot acetone and ethanol baths for 10 minutes each before using.

We transferred graphene onto the front substrate of the flow cell following a wet transfer procedure [1, 2]. First, we spin-coated graphene samples with CAB at 4000 rpm and baked them at 180°C for 3 minutes. Then, to remove unnecessary graphene on the backside of copper substrates, the CAB-coated graphene samples were immersed into a 1 M solution of APS for 10 minutes and subsequently rinsed with MilliQ water five times. The copper substrates were then fully etched by 0.1 M APS solution for 2 hours, followed by a five times rinse with MilliQ water to remove the attached ions. Then, the floating CAB-graphene monolayers were "fished" onto the flow cell, and the CAB coating was removed by soaking in acetone for 2 hours and in isopropanol for one hour.

1.2 OPTP measurements

We probed electron relaxation in graphene using optical pump - terahertz probe (OPTP) spectroscopy. A schematic of the OPTP setup is shown in Fig. S1. The fundamental laser output was generated by a regenerative Ti:sapphire amplifier system, which produces 5 W, 50 fs pulses at a repetition rate of 1 kHz and a central wavelength of 800 nm. The generated pulses were then split into three branches for THz generation, sampling, and optical excitation. A single-cycle THz pulse of ∼ 1 ps duration was generated by pumping a 1 mm thick (110) ZnTe crystal with the 800 nm fundamental pulses via optical rectification.

We photoexcited graphene to generate hot carriers by using 800 nm pulses with a diameter of

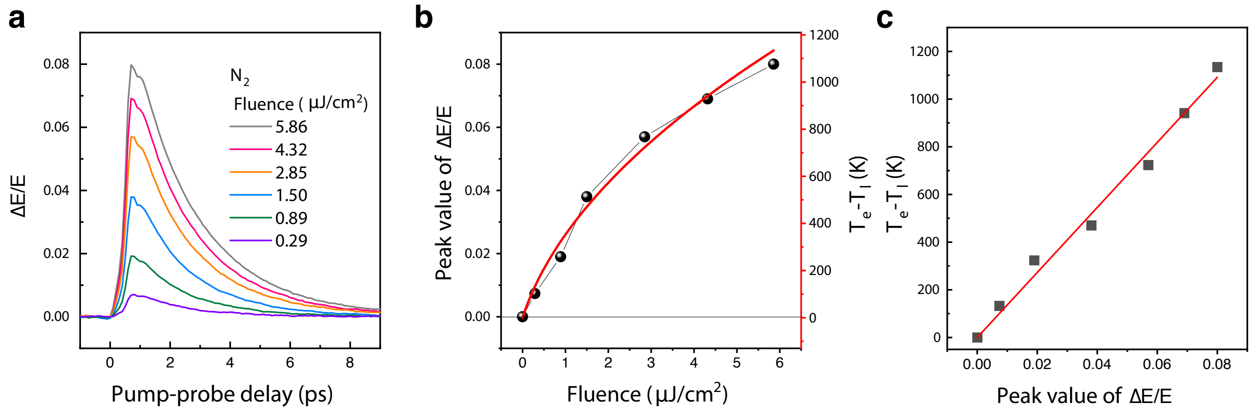

FIGURE S2: **Electron temperature of the pumped graphene layer**. **a**.The OPTP traces of graphene in a nitrogen atmosphere with various excitation fluences. **b**. Peak value of $\Delta E/E$ as a function of laser fluence and corresponding electron temperature. **c**. Increase in electron temperature (T_e) with respect to ambient temperature T_ℓ as a function of $\Delta E/E$: a linear relation is observed.

5 mm to ensure a homogeneously photoexcited region. The transmitted THz wave was then recollimated and focused onto a ZnTe detection crystal together with an 800 nm sampling beam, where the THz electrical field waveform was detected using the electro-optic sampling method [3]. The THz pulse induces birefringence in the ZnTe detection crystal, and the polarization of the sampling beam is thus changed. After passing through a quarter-wave plate, the sampling beam changes from perfectly circular to slightly elliptical shape. The s and p components of this elliptically polarized pulse are separated by a Wollaston prism, and the difference of these two components is detected by a balance diode. The signal is collected by a lock-in amplifier that is phase-locked to an optical chopper that modulates either the THz generation beam or the pump beam at a frequency of 500 Hz. The ultrafast time evolution of the peak intensity of the THz field is tracked by varying the time delay between optical pump and THz probe [3, 4]. The setup was purged with dry nitrogen during the measurement to avoid the absorption of water vapor.

The raw data consists in time traces of the pump-induced transmission change at the peak of the THz waveform (ΔE), normalized by the peak value of the THz transmission without excitation (E) (Fig. S2a). Assuming that a fraction $\gamma = 1.6\%$ of the pump pulse energy is absorbed by the graphene electrons [5], the maximum electron temperature reached after photoexcitation can be related to the pump laser fluence F according to $\gamma F = C(T_\mathrm{e})T_\mathrm{e}$, where $C(T_\mathrm{e})$ is the graphene heat capacity at temperature T_e. In the limit where the graphene Fermi energy μ is larger than $k_\mathrm{B}T_\mathrm{e}$ (as relevant for our samples), we may use the approximate expression [6, 7, 8]

$$C(T_\mathrm{e}) = \alpha T_\mathrm{e}, \quad \text{with} \quad \alpha = \frac{2\pi}{3}\frac{k_\mathrm{B}^2\mu}{(\hbar v_\mathrm{F})^2}, \tag{1}$$

where v_F is graphene's constant Fermi velocity. Then,

$$T_\mathrm{e} = T_0\left(1 + \frac{2\gamma F}{\alpha T_0^2}\right)^{1/2}, \tag{2}$$

where T_0 is ambient temperature. The peak value of $\Delta E/E$ after photoexcitation increases with laser fluence. Upon rescaling, we find that the plots of $\Delta E/E$ vs. F and T_e vs. F collapse upon each other (Fig. S2b), so that we may consider that $\Delta E/E$ is proportional to the electron temperature within the range of temperatures probed in the experiment, as shown explicitly in Fig. S2c.

The thickness of the liquid layer was set to 50 μm by the geometry of the flow cell. The liquids were exchanged using a syringe and the spectroscopic measurement was always carried out at

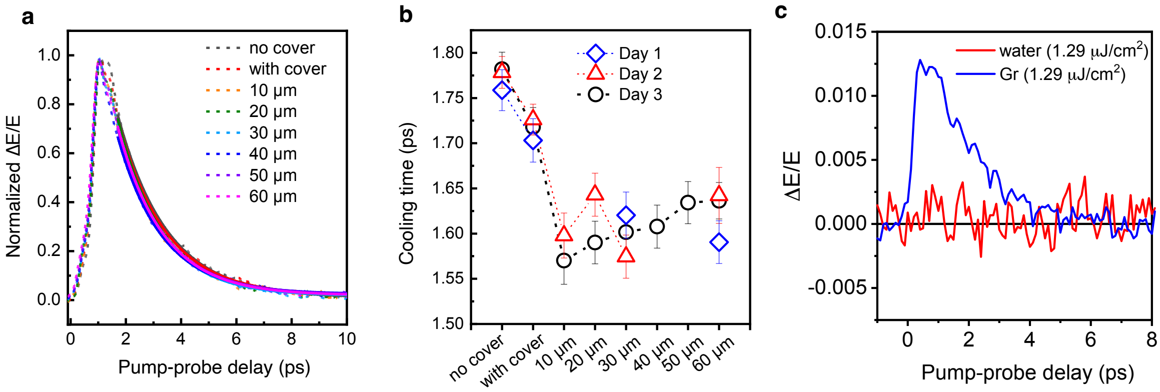

FIGURE S3: **Control experiments**. **a**. The OPTP traces of graphene with varying water layer thickness. **b**. Cooling times obtained by exponential fitting of the data in panel a. The error bars represent 95% confidence intervals of the exponential fits. **c**. Comparison of the THz signals after photoexcitation, with and without graphene. Water in the absence of graphene shows no measurable THz response.

the same spot of the graphene sample. To exclude the effect of beam dispersion in the different liquids on the results, we repeated the measurement with different water layer thickness and using different Teflon spacers between two fused silica windows (Fig. S3 a and b). We further checked that there was no THz signal from water in the absence of graphene (Fig. S3c).

1.3 FTIR measurements

We measured the dielectric functions of water, heavy water, ethanol and methanol using Fourier-transform infrared (FTIR) spectroscopy. We measured the transmitted and reflected infrared intensities both for an empty cell ($I_{\rm t,cell}, I_{\rm r,cell}$) and for a cell filled with liquid ($I_{\rm t,liquid}, I_{\rm r,liquid}$) thanks to an A510/Q-T Reflectance and Transmittance accessory placed in a commercial VERTEX 70 FTIR spectrometer (Fig. S4a). In order to avoid disassembling the cell when changing liquids, we carried out the measurements inside a flow cell, made out of two-silicon wafers separated by a 10 μm Teflon spacer.

We calculated the absorbance $A(\omega)$ according to

$$A(\omega) = -\log_{10}\left(\frac{I_{\rm t,solution}(\omega)}{I_{\rm t,cell}(\omega) + I_{\rm r,cell}(\omega) - I_{\rm r,solution}(\omega)}\right). \tag{3}$$

The H_2O and D_2O show saturated absorption in the range of 3100-3600 and 2200-2700 cm^{-1}, respectively. We obtained the data in this frequency range by measuring the spectra without any spacer between two CaF_2 windows and then rescaled the spectra to overlap with the data with spacer (Fig. S4b). The imaginary part $k(\omega)$ of the refractive index is related to the absorbance by

$$k(\omega) = A(\omega)\frac{\ln(10)}{4\pi\omega\ell}, \tag{4}$$

where ℓ is the sample thickness. To accurately determine the thickness of the cell, we calculate the absorbance of the empty cell without correction for multiple reflections,

$$A_2(\omega) = -\log_{10}\left(\frac{I_{\rm t,cell}(\omega)}{I_{\rm t,lamp}(\omega)}\right). \tag{5}$$

where $I_{\rm t,lamp}(\omega)$ is the intensity of the lamp of the FTIR source (Fig. S5a). Fourier transformation of this spectrum yields a peak at the time Δt that light takes to travel twice through the cell (Fig.

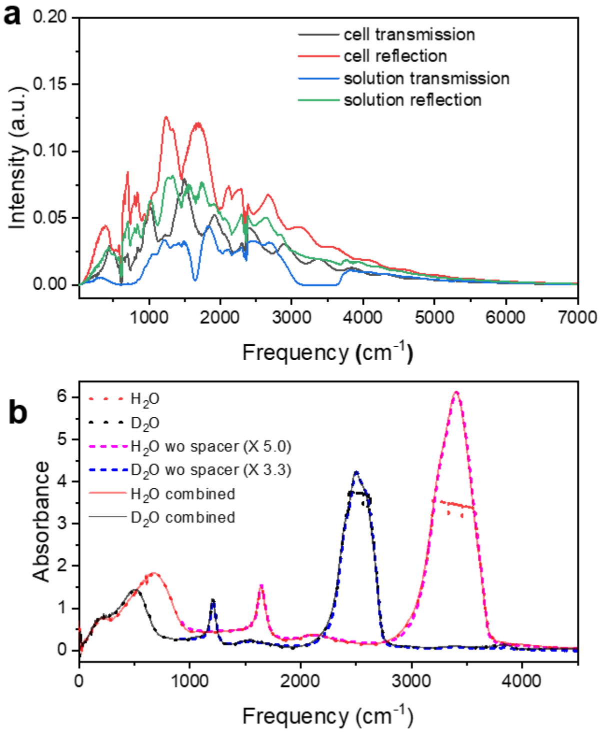

FIGURE S4: **FTIR data analysis**. **a**. Raw intensity data of empty cell and water-filled cell. **b**. Absorbance of H_2O and D_2O, measured with spacer and without spacer (the latter is rescaled to overlap with the former).

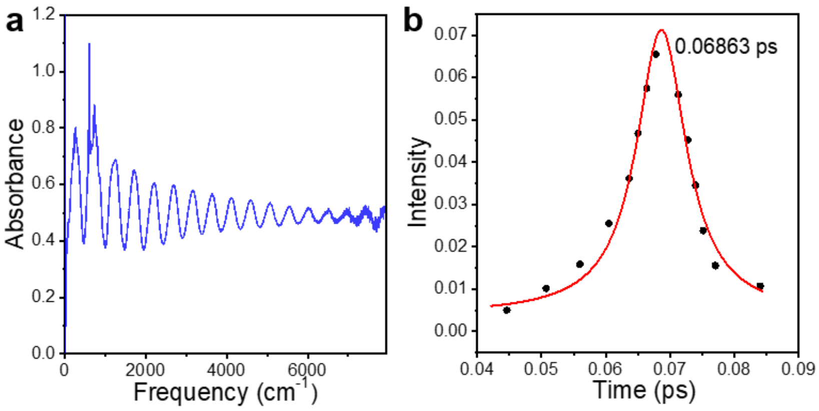

FIGURE S5: **Determination of the cell thickness**. **a**. Absorbance of empty cell. **b**. Fourier transform of the data in panel a.

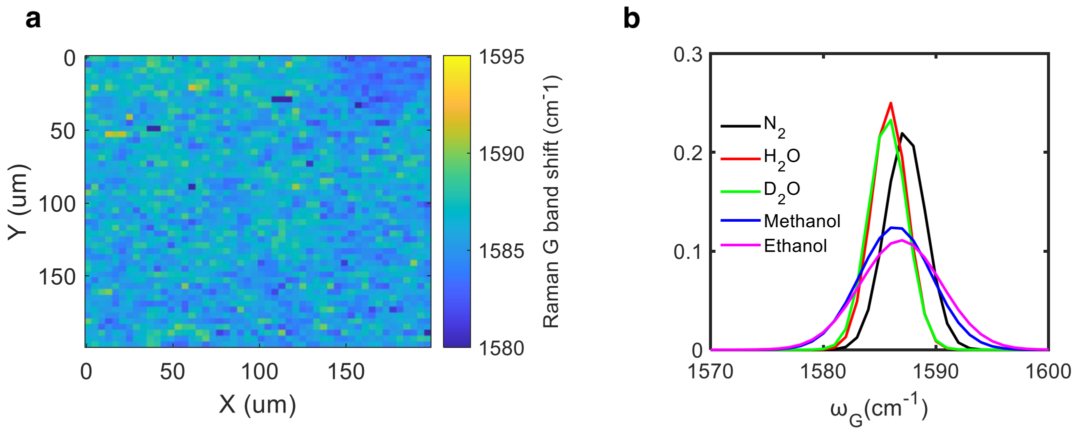

FIGURE S6: **Raman characterization of graphene sample**. **a**. Spatial map of Raman G band frequency for graphene sample in air. **b**. Distribution of the Raman G band frequency with different liquids placed on the graphene surface.

S5b), so that $\ell = c\Delta t/2 = 10.29\ \mu$m. We then obtained the real part of the refractive index through a numerical Kramers-Krönig transformation:

$$n(\omega) = n_\infty + \frac{2}{\pi}\int_0^\infty \mathrm{d}\omega' \, \frac{k(\omega')}{\omega' - \omega}, \tag{6}$$

where n_∞ is the refractive index in the high frequency limit, which is obtained by the ATAGO Digital Handheld Refractometer: PAL-RI. The measured values for H_2O, D_2O, methanol, ethanol and isopropanol are 1.333, 1.3291, 1.3285, 1.3604, and 1.3706 respectively.

We then obtain the dielectric function $\epsilon(\omega) = \epsilon'(\omega) + i\epsilon''(\omega)$ according to

$$\begin{cases} \epsilon'(\omega) = n(\omega)^2 - k(\omega)^2 \\ \epsilon''(\omega) = 2n(\omega)k(\omega) \end{cases}. \tag{7}$$

1.4 Raman measurements

We estimated the Fermi level μ in our liquid-covered graphene samples from the Raman G-band frequency, according to the empirical equation [6]

$$|\mu|(\mathrm{eV}) = \frac{\omega_\mathrm{G} - 1580\ \mathrm{cm}^{-1}}{42\ \mathrm{cm}^{-1}}. \tag{8}$$

An example of a spatial map of the Raman G-band frequency is shown in Fig. S6a. The frequency shows spatial inhomogeneities on the μm scale with an amplitude around 10 cm^{-1}. The corresponding distributions are shown in Fig. S6b. The average G-band frequency is essentially independent of the nature of the liquid, which excludes a change in charge carrier density as a possible mechanism for the liquid effect on the electron cooling rate. To take into account the broadness of the distribution, in the theoretical analysis we considered chemical potentials in the range $\mu = 100 - 180$ meV. The theoretical prediction is independent of the electron or hole nature of the charge carriers.

2 Theoretical methods

In this section, we develop a description of energy transfer between the Dirac fermion charge carriers in graphene and a liquid, treated as a bosonic bath, within the non-equilibrium Keldysh framework of perturbation theory. For the sake of completeness, and in order to show consistency

with previous theoretical approaches, we apply the same description to energy transfer between the graphene electrons and its optical phonon modes, showing that our formalism recovers the results that were previously obtained within a Boltzmann equation approach [9].

We use SI units throughout the text. We adopt the following convention for the n-dimensional Fourier transform:

$$\tilde{f}(\mathbf{q}) = \int_{-\infty}^{+\infty} \mathrm{d}^n\mathbf{r}\, f(\mathbf{r}) e^{-i\mathbf{q}\cdot\mathbf{r}} \quad \text{and} \quad f(\mathbf{r}) = \frac{1}{(2\pi)^n}\int_{-\infty}^{+\infty} \mathrm{d}^n\mathbf{q}\, \tilde{f}(\mathbf{q}) e^{i\mathbf{q}\cdot\mathbf{r}}. \tag{9}$$

2.1 Interaction Hamiltonian

2.1.1 Electron-hydron interaction

In this section, $\mathbf{r}$ *represents a vector in 3D space, and* $\boldsymbol{\rho}$ *a vector in 2D space.* The charge fluctuations of the liquid in the $z > 0$ half-space couple to the graphene electrons via the Coulomb potential V. In real space, the corresponding Hamiltonian is

$$H_{\mathrm{ew}}(t) = \int \mathrm{d}\mathbf{r}\mathrm{d}\mathbf{r}' n_w(\mathbf{r},t) V(\mathbf{r}-\mathbf{r}') n_e(\mathbf{r},t), \tag{10}$$

where n_w and n_e are the liquid and graphene instantaneous charge density, respectively. Let $c^\dagger_{\mathbf{k},\nu}, c_{\mathbf{k},\nu}$ be the Dirac fermion creation and annihilation operators in the chiral basis ($\nu = \pm 1$). A 2D Fourier transformation then yields

$$H_{\mathrm{int}} = \int \frac{\mathrm{d}\mathbf{q}}{(2\pi)^2}\frac{e^2}{2\epsilon_0 q} n_{\mathrm{s}}(q,t) \sum_{\mathbf{k},\nu,\nu'} \langle \mathbf{k}+\mathbf{q},\nu| e^{i\mathbf{q}\boldsymbol{\rho}} e^{qz} |\mathbf{k},\nu'\rangle c^\dagger_{\mathbf{k}+\mathbf{q},\nu}(t) c_{\mathbf{k},\nu'}(t), \tag{11}$$

with

$$n_{\mathrm{s}}(q) = \int \mathrm{d}\boldsymbol{\rho} \int_0^{+\infty} \mathrm{d}z\, e^{-i\mathbf{q}\boldsymbol{\rho}} e^{-qz} n_{\mathrm{w}}(\boldsymbol{\rho}, z, t). \tag{12}$$

As long as we consider wavevectors $\mathbf{q}$ such that q^{-1} is large compared to the extension of the carbon p_z orbitals perpendicular to the graphene plane, we may approximate

$$|\langle \mathbf{k}+\mathbf{q},\nu| e^{i\mathbf{q}\boldsymbol{\rho}} e^{qz} |\mathbf{k},\nu'\rangle|^2 \approx |\langle \mathbf{k}+\mathbf{q},\nu| e^{i\mathbf{q}\boldsymbol{\rho}} |\mathbf{k},\nu'\rangle|^2 = \frac{1}{2}\left(1 + \nu\nu' \cos(\phi_{\mathbf{k}+\mathbf{q}} - \phi_{\mathbf{q}})\right). \tag{13}$$

2.1.2 Electron-phonon interaction

Let $d^\dagger_{\mathbf{q},\alpha}, d_{\mathbf{q},\alpha}$ be the creation and annihilation operators of phonons in the mode α with frequency ω_α. The non-interacting electron-phonon system's Hamiltonian is

$$H_0 = \sum_{\mathbf{k},\nu} E_{\mathbf{k},\nu} c^\dagger_{\mathbf{k},\nu} c_{\mathbf{k},\nu} + \sum_{\mathbf{q},\alpha} \hbar\omega_\alpha d^\dagger_{\mathbf{q},\alpha} d_{\mathbf{q},\alpha}, \tag{14}$$

where $E_{\mathbf{k},\nu}$ are the band energies, and $\sum_{\mathbf{k}} \equiv (1/\mathcal{A}_{\mathrm{BZ}}) \int_{\mathrm{BZ}} \mathrm{d}\mathbf{k}$ ($\mathcal{A}_{\mathrm{BZ}}$ is the area of the 2D Brillouin zone). The electron-phonon interaction Hamiltonian has the general form [10]

$$H_{\mathrm{ep}} = \sum_\alpha \int_{\mathrm{BZ}} \frac{\mathrm{d}\mathbf{q}}{(2\pi)^2} \sum_{\mathbf{k},\nu,\nu'} g^{\nu\nu'}_{\alpha,\mathbf{k},\mathbf{k}+\mathbf{q}} c^\dagger_{\mathbf{k}+\mathbf{q}} c_{\mathbf{k}} (d^\dagger_{\mathbf{q},\alpha} + d_{-\mathbf{q},\alpha}), \tag{15}$$

Following [9], we consider the Γ point LO and TO phonons that scatter electrons within one valley, and the K, K' point LO phonons that scatter electrons between valleys. The electron-phonon matrix elements read

$$|g^{\nu\nu'}_{\Gamma,\mathbf{k},\mathbf{k}+\mathbf{q}}|^2 = g_\Gamma^2 (1 \pm \nu\nu' \cos(\phi_{\mathbf{k}} + \phi_{\mathbf{k}+\mathbf{q}} - 2\phi_{\mathbf{q}})), \tag{16}$$

where the $+$ ($-$) sign is for LO (TO) phonons; and

$$|g^{\nu\nu'}_{\Gamma,\mathbf{k},\mathbf{k}+\mathbf{q}}|^2 = g^2_{\mathrm{K}}(1 \mp \nu\nu' \cos(\phi_{\mathbf{k}} - \phi_{\mathbf{k}+\mathbf{q}})), \tag{17}$$

where the $-$ ($+$) sign corresponds to scattering from K to K' (from K' to K); here, $\phi_{\mathbf{v}}$ is the polar angle of the vector $\mathbf{v}$. The values of the coupling constants are $g_\Gamma = 0.55$ eV·Å and $g_{\mathrm{K}} = 0.85$ eV·Å, according to GW calculations [11].

2.1.3 General form

We find that for both types of interactions the Hamiltonian has the general form

$$H_{\mathrm{eb}} = \int \frac{\mathrm{d}\mathbf{q}}{(2\pi)^2} n_{\mathbf{q}}(t)\varphi_{\mathbf{q}}(t), \tag{18}$$

where $n_{\mathbf{q}}$ is an electronic two-particle operator and $\varphi_{\mathbf{q}}$ is a free bosonic field. In the electron-phonon case, we define

$$n_{\mathbf{q}} = \sum_{\mathbf{k},\nu,\nu'} \frac{g^{\nu\nu'}_{\alpha,\mathbf{k},\mathbf{k}+\mathbf{q}}}{\sqrt{\hbar\omega_\alpha}} c^\dagger_{\mathbf{k}+\mathbf{q},\nu} c_{\mathbf{k},\nu'} \quad \text{and} \quad \varphi_{\mathbf{q}} = \sqrt{\hbar\omega_\alpha}(d^\dagger_{\mathbf{q},\alpha} + d_{-\mathbf{q},\alpha}); \tag{19}$$

in the electron-hydron case

$$n_{\mathbf{q}} = \sqrt{V_q} \sum_{\mathbf{k},\nu,\nu'} \langle \mathbf{k}+\mathbf{q},\nu | e^{i\mathbf{q}\boldsymbol{\rho}} | \mathbf{k},\nu'\rangle c^\dagger_{\mathbf{k}+\mathbf{q},\nu} c_{\mathbf{k},\nu'} \quad \text{and} \quad \varphi_{\mathbf{q}} = \sqrt{V_q} n_{\mathrm{s}}(\mathbf{q}), \tag{20}$$

where $V_q \equiv e^2/(2\epsilon_0 q)$ is the 2D Fourier-transformed Coulomb potential. With these definitions, both $n_{\mathbf{q}}$ and $\phi_{\mathbf{q}}$ have dimensionless correlation functions in frequency space.

2.2 General theory of electron-boson heat transfer

2.2.1 Non-equilibrium perturbation theory

We consider an initial state of the electron-boson system where the electrons are at a temperature T_{e} and the bosons at a temperature T_{b}. We wish to study the subsequent dynamics. In particular, we are interested in the heat flux per unit surface from the electrons to the bosons:

$$\mathcal{Q}(t) = -\frac{1}{\mathcal{A}} \frac{\mathrm{d}}{\mathrm{d}t} \langle H_{\mathrm{eb}}(t) \rangle. \tag{21}$$

Since the system is under non-equilibrium conditions, this average value needs to be computed in the Keldysh framework. In particular, we may define the Keldysh component of the electron-boson correlation function:

$$\chi^K_{\mathrm{eb}}(\mathbf{q},t,t') = -\frac{1}{\mathcal{A}} \frac{i}{\hbar} \langle \{ n_{\mathbf{q}}(t), \varphi_{-\mathbf{q}}(t') \} \rangle. \tag{22}$$

Then,

$$\mathcal{Q}(t) = -\frac{i\hbar}{2} \int \frac{\mathrm{d}\mathbf{q}}{(2\pi)^2} \frac{\mathrm{d}\chi^K_{\mathrm{eb}}(\mathbf{q},t,t)}{\mathrm{d}t}. \tag{23}$$

Form this point on, the computation of the electron-boson correlation function follows the exact same steps as in the theory of quantum friction [12], and we reproduce here only the main equations. Diagramatically, the correlation function satisfies the following Dyson equation:

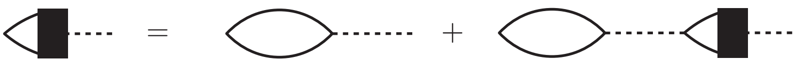

(24)

where the "bubble" represents the propagator of n (denoted χ_e), and the dashed line the propagator of φ (denoted χ_b). When made explicit in terms of the R, A, K components, the Dyson equation becomes

$$\begin{cases} \chi_{\text{eb}}^K = \chi_{\text{e}}^R \otimes \chi_{\text{b}}^K + \chi_{\text{e}}^K \otimes \chi_{\text{b}}^A + \chi_{\text{e}}^R \otimes \chi_{\text{b}}^R \otimes \chi_{\text{eb}}^K + (\chi_{\text{e}}^R \otimes \chi_{\text{b}}^K + \chi_{\text{e}}^K \otimes \chi_{\text{b}}^A) \otimes \chi_{\text{eb}}^A \\ \chi_{\text{eb}}^{R,A} = \chi_{\text{e}}^{R,A} \otimes \chi_{\text{b}}^{R,A} + \chi_{\text{e}}^{R,A} \otimes \chi_{\text{b}}^{R,A} \otimes \chi_{\text{eb}}^{R,A} \end{cases}, \tag{25}$$

where $\otimes$ represents time convolution. While these equations are extremely general, they are impractical to manipulate analytically, unless a number of assumptions are made. In order to proceed, we will restrict ourselves to cooling dynamics that are slow enough for time-translation invariance to hold when it comes to determining the cooling rate. This assumption is expected to hold for small enough temperature differences, such that the cooling rate is approximately temperature-independent. We will further assume that, in line with experimental observations, that electron thermalization is much faster than electron-boson energy transfer, so that the electron and boson propagators may be considered as equilibrium propagators, satisfying the fluctuation-dissipation theorem: we work within a two-temperature model. We may then carry out Fourier transforms in time, so that Eq. (23) becomes

$$\mathcal{Q} = \frac{1}{2} \int \frac{\mathrm{d}\mathbf{q}\mathrm{d}\omega}{(2\pi)^3} \hbar\omega\, \chi_{\text{eb}}^K(\mathbf{q}, \omega). \tag{26}$$

The convolutions in Eq. (25) become products in Fourier space. Before proceeding, it is convenient to flip the signs of all the correlation functions: we introduce, for all the labels, $g \equiv -\chi$. Then, after some algebra, we obtain an explicit expression for $\mathcal{Q}$:

$$\mathcal{Q} = \frac{1}{2\pi^3} \int \mathrm{d}\mathbf{q} \int_0^{+\infty} \mathrm{d}\omega\, \hbar\omega [n_{\text{B}}(\omega, T_{\text{e}}) - n_B(\omega, T_{\text{b}})] \frac{\text{Im}\,[g_{\text{e}}^R(\mathbf{q}, \omega)]\text{Im}\,[g_{\text{b}}^R(\mathbf{q}, \omega)]}{|1 - g_{\text{e}}^R(\mathbf{q}, \omega) g_{\text{b}}^R(\mathbf{q}, \omega)|^2}, \tag{27}$$

where $n_{\text{B}}(\omega, T) \equiv 1/(e^{\hbar\omega/k_B T} - 1)$ is the Bose distribution at temperature T. We recover Eq. (3) of the main text.

2.2.2 Cooling rate

The cooling dynamics are governed by the equation

$$\frac{\mathrm{d}\mathcal{E}(T_{\text{e}})}{\mathrm{d}t} = -\mathcal{Q}(T_{\text{e}}, T_{\text{b}}), \tag{28}$$

where $\mathcal{E}$ is the total energy per unit surface of the electronic system. We follow ref. [9] in determining the electronic heat capacity (per unit surface) at constant density $C(T_{\text{e}})$, such that $\mathrm{d}_t\mathcal{E} = C(T_{\text{e}})\mathrm{d}_t T_{\text{e}}$. We may then define the instantaneous cooling rate

$$\tau(T_{\text{e}}, T_{\text{b}}) = \frac{C(T_{\text{e}})(T_{\text{e}} - T_{\text{b}})}{\mathcal{Q}(T_{\text{e}}, T_{\text{b}})}. \tag{29}$$

2.3 Application to the graphene-liquid system

2.3.1 Liquid-mediated cooling

We first consider electron cooling through the electron-hydron coupling. Using eqs. (12) and (20), we find that

$$g_{\text{b}}^R(\mathbf{q}, t, t') = -\frac{1}{\mathcal{A}} V_q \int_0^{+\infty} \mathrm{d}z\mathrm{d}z'\, e^{-q(z+z')} \left[-\frac{i}{\hbar}\theta(t - t')\langle [n_{\text{s}}(\mathbf{q}, z, t), n_{\text{s}}(-\mathbf{q}, z', t')] \rangle \right]. \tag{30}$$

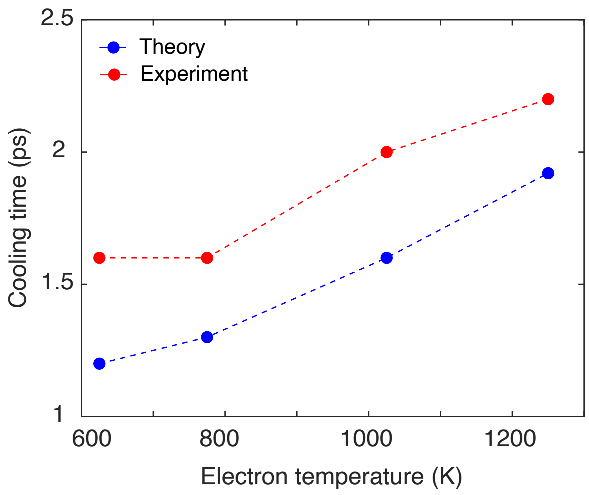

FIGURE S7: Dependence of water-mediated cooling time on initial electron temperature. The red dots are experimental data for graphene in contact with water and the red dots correspond to the prediction of Eq. (29) (with $\mu = 180$ meV).

This is the microscopic definition of the liquid's *surface response function*. In the long wavelength limit, it can be expressed in terms of the liquid's bulk dielectric function $\epsilon(\omega)$ [12]:

$$g_{\mathrm{b}}^{R}(\mathbf{q},\omega) = \frac{\epsilon(\omega)-1}{\epsilon(\omega)+1}, \tag{31}$$

as stated in the main text. The electronic response function $g_{\mathrm{e}}^{R}(\mathbf{q},\omega)$ simply amount to (minus) the density-density response function. Taking into account electron-electron interactions at the RPA level [13],

$$g_{\mathrm{e}}^{R}(q,\omega) = -\frac{V_q \chi_{\mathrm{e}}^{0}(q,\omega)}{1 - V_q \chi_{\mathrm{e}}^{0}(q,\omega)}. \tag{32}$$

The non-interacting response function χ_e^0 is given by [13]

$$\chi_{\mathrm{e}}^{0}(q,\omega) = g_{\mathrm{s}} g_{\mathrm{v}} \int \frac{\mathrm{d}\mathbf{k}}{(2\pi)^2} \sum_{\nu,\nu'} |\langle \mathbf{k}+\mathbf{q},\nu | e^{i\mathbf{q}\boldsymbol{\rho}} | \mathbf{k},\nu' \rangle|^2 \frac{n_{\mathrm{F}}(E_{\mathbf{k}}^{\nu},T_{\mathrm{e}}) - n_{\mathrm{F}}(E_{\mathbf{k+q}}^{\nu'},T_{\mathrm{e}})}{E_{\mathbf{k}}^{\nu} - E_{\mathbf{k+q}}^{\nu'} + \omega + i\delta}, \tag{33}$$

where $g_{\mathrm{s}} = g_{\mathrm{v}} = 2$ are the spin and valley degeneracies of graphene, respectively, $E_{\mathbf{k}}^{\nu} = \nu v_F k$ are the band energies in the Dirac fermion approximation, $n_{\mathrm{F}}(E,T) = 1/(e^{(E-\mu)/k_B T}+1)$ is the Fermi distribution at chemical potential μ and temperature T, and $\delta \to 0^+$. The integral is evaluated numerically at non-zero temperature.

With all the above, we may compute theoretical predictions for the liquid-mediated cooling rate by numerical integration according to Eq. (27). We considered a graphene chemical potential μ in the range $100-180$ meV (see section 1.4) and an electron temperature $T_{\mathrm{e}} = 623$ K, corresponding to the lowest pump laser fluence. Our model is further able to reproduce the dependence of the electron cooling time on T_{e}, as shown in Fig. S7.

We note that Eq. (27) involves bare surface response functions, that contain no effect of the presence of the neighboring medium, at least at the RPA level. Nevertheless, the physical response function of graphene in the presence of water undergoes RPA renormalization according to

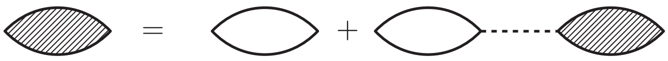

(34)

In this diagrammatic equation, when the propagators are interpreted as surface response functions, the vertices reduce to unity, so that we obtain the renormalized graphene response function $\tilde{g}_{\mathrm{e}}$ as

$$\tilde{g}_{\mathrm{e}}(q,\omega) = \frac{g_{\mathrm{e}}(q,\omega)}{1 - g_{\mathrm{e}}(q,\omega) g_{\mathrm{b}}(q,\omega)}, \tag{35}$$

which is Eq. (7) of the main text.

2.3.2 Phonon-mediated cooling

In the phonon case, the boson response function is proportional to the usual phonon propagator:

$$g_{\rm b}^R(\mathbf{q},\omega) = \frac{2\omega_\alpha^2}{\omega_\alpha^2 - \omega^2}. \tag{36}$$

The non-interacting electronic response function now involves the electron-phonon matrix elements:

$$g_{\rm e}^R(q,\omega) = -g_s \int_{\rm BZ} \frac{{\rm d}\mathbf{k}}{(2\pi)^2} \sum_{\nu,\nu'} \frac{|g^{\nu\nu'}_{\alpha,\mathbf{k},\mathbf{k}+\mathbf{q}}|^2}{\hbar\omega_\alpha} \frac{n_{\rm F}(E^\nu_{\mathbf{k}},T_{\rm e}) - n_{\rm F}(E^{\nu'}_{\mathbf{k}+\mathbf{q}},T_{\rm e})}{E^\nu_{\mathbf{k}} - E^{\nu'}_{\mathbf{k}+\mathbf{q}} + \omega + i\delta}. \tag{37}$$

We now show that we recover the results of ref. [9] for the electron-phonon cooling rate obtained in a Boltzamann equation framework, if we neglect electron-electron interactions and treat electron-phonon interactions to first order. Under these assumptions, Eq. (27) reduces to

$$\mathcal{Q} = \frac{1}{2\pi^3} \int {\rm d}\mathbf{q} \int_0^{+\infty} {\rm d}\omega\, \hbar\omega [n_{\rm B}(\omega,T_{\rm e}) - n_{\rm B}(\omega,T_{\rm b})] {\rm Im}\,[g_{\rm e}^R(\mathbf{q},\omega)] {\rm Im}\,[g_{\rm b}^R(\mathbf{q},\omega)]. \tag{38}$$

We notice that

$$ {\rm Im}\,[g_{\rm b}^R(q,\omega)] = \pi\omega_\alpha^2[\delta(\omega-\omega_\alpha) - \delta(\omega+\omega_\alpha)] \tag{39}$$

and

$${\rm Im}\,[g_{\rm e}^R(\mathbf{q},\omega)] = \pi g_s \int_{\rm BZ} \frac{{\rm d}\mathbf{k}}{(2\pi)^2} \sum_{\nu,\nu'} \frac{|g^{\nu\nu'}_{\alpha,\mathbf{k},\mathbf{k}+\mathbf{q}}|^2}{\hbar\omega_\alpha} [n_{\rm F}(E^\nu_{\mathbf{k}},T_{\rm e}) - n_{\rm F}(E^{\nu'}_{\mathbf{k}+\mathbf{q}},T_{\rm e})]\delta(E^\nu_{\mathbf{k}} - E^{\nu'}_{\mathbf{k}+\mathbf{q}} + \omega). \tag{40}$$

Moreover, upon integration over $\mathbf{k}$ and $\mathbf{q}$ in Eq. (38), the angle-dependent parts of the electron-phonon matrix elements vanish, and the intervalley phonons become formally identical to the intravalley phonons: we may introduce the valley degeneracy and carry out integrations over a single Dirac cone. Altogether, we obtain

$$\begin{aligned} \mathcal{Q} = 2\pi g_s g_v \omega_\alpha g_\alpha^2 [n_{\rm B}(\omega_\alpha,T_{\rm e}) - n_{\rm B}(\omega_\alpha,T_{\rm b})] \dots \\ \dots \sum_{\nu,\nu'} \int \frac{{\rm d}\mathbf{q}{\rm d}\mathbf{k}}{(2\pi)^4} [n_{\rm F}(E^\nu_{\mathbf{k}},T_{\rm e}) - n_{\rm F}(E^{\nu'}_{\mathbf{q}},T_{\rm e})]\delta(E^\nu_{\mathbf{k}} - E^{\nu'}_{\mathbf{q}} + \omega_\alpha). \end{aligned} \tag{41}$$

If we introduce another delta function, according to

$$\begin{aligned} \mathcal{Q} = 2\pi g_s g_v \omega_\alpha g_\alpha^2 [n_{\rm B}(\omega_\alpha,T_{\rm e}) - n_{\rm B}(\omega_\alpha,T_{\rm b})] \dots \\ \dots \sum_{\nu,\nu'} \int \frac{{\rm d}\mathbf{q}{\rm d}\mathbf{k}}{(2\pi)^4} \int_{-\infty}^{+\infty} {\rm d}\epsilon [n_{\rm F}(\epsilon-\omega_\alpha,T_{\rm e}) - n_{\rm F}(\epsilon,T_{\rm e})]\delta(E^\nu_{\mathbf{k}} - \epsilon + \omega_\alpha)\delta(\epsilon - E^{\nu'}_{\mathbf{q}}), \end{aligned} \tag{42}$$

we recognize the graphene density of states,

$$\overline{\nu}(\epsilon) = g_s g_v \sum_\nu \int \frac{{\rm d}\mathbf{k}}{(2\pi)^2} \delta(\epsilon - E_{\mathbf{k},\nu}) = \frac{2|\epsilon|}{\pi v_F^2}. \tag{43}$$

Our result then simplifies according to

$$\mathcal{Q} = \frac{2\pi\omega_\alpha g_\alpha^2}{g_s g_v} [n_{\rm B}(\omega_\alpha,T_{\rm e}) - n_{\rm B}(\omega_\alpha,T_{\rm b})] \int_{-\infty}^{+\infty} {\rm d}\epsilon [n_{\rm F}(\epsilon-\omega_\alpha,T_{\rm e}) - n_{\rm F}(\epsilon,T_{\rm e})]\overline{\nu}(\epsilon)\overline{\nu}(\epsilon-\omega_\alpha), \tag{44}$$

which is Eq. (18) in the supplementary information of ref. [9].

References

[1] Yogeswaran, N. *et al.* Piezoelectric graphene field effect transistor pressure sensors for tactile sensing. *Applied Physics Letters* **113**, 014102 (2018).

[2] Burwell, G., Smith, N. & Guy, O. Investigation of the utility of cellulose acetate butyrate in minimal residue graphene transfer, lithography, and plasma treatments. *Microelectronic Engineering* **146**, 81–84 (2015).

[3] Ulbricht, R., Hendry, E., Shan, J., Heinz, T. F. & Bonn, M. Carrier dynamics in semiconductors studied with time-resolved terahertz spectroscopy. *Reviews of Modern Physics* **83**, 543–586 (2011).

[4] Lee, Y.-S. *Principles of Terahertz Science and Technology* (Springer US, 2009).

[5] Fu, S. *et al.* Long-lived charge separation following pump-wavelength-dependent ultrafast charge transfer in graphene/ws_2 heterostructures. *Science Advances* **7**, eabd9061 (2021).

[6] Shi, S. F. *et al.* Controlling graphene ultrafast hot carrier response from metal-like to semiconductor-like by electrostatic gating. *Nano Letters* **14**, 1578–1582 (2014).

[7] Tielrooij, K. J. *et al.* Photoexcitation cascade and multiple hot-carrier generation in graphene. *Nature Physics* **9**, 248–252 (2013).

[8] Lui, C. H., Mak, K. F., Shan, J. & Heinz, T. F. Ultrafast photoluminescence from graphene. *Physical Review Letters* **105**, 127404 (2010).

[9] Pogna, E. A. *et al.* Hot-carrier cooling in high-quality graphene is intrinsically limited by optical phonons. *ACS Nano* **15**, 11285–11295 (2021).

[10] Neto, A. H. C. & Guinea, F. Electron-phonon coupling and raman spectroscopy in graphene. *Physical Review B* **75**, 045404 (2007).

[11] Sohier, T. *et al.* Phonon-limited resistivity of graphene by first-principles calculations: Electron-phonon interactions, strain-induced gauge field, and boltzmann equation. *Physical Review B* **90**, 125414 (2014).

[12] Kavokine, N., Bocquet, M.-L. & Bocquet, L. Fluctuation-induced quantum friction in nanoscale water flows. *Nature* **602**, 84–90 (2022).

[13] Wunsch, B., Stauber, T., Sols, F. & Guinea, F. Dynamical polarization of graphene at finite doping. *New Journal of Physics* **8**, 318–318 (2006).
